# Incidence and Risk Factors of Intraventricular Hemorrhage in Early Preterm Infants: A Cross-Sectional Study

**DOI:** 10.7759/cureus.68500

**Published:** 2024-09-03

**Authors:** Seema Sharafat, Zahid Khan, Amir Muhammad, Haidar Ali, Adnan Khan, Ahmad Noushad

**Affiliations:** 1 Neurosurgery, Lady Reading Hospital Medical Teaching Institution (MTI) Peshawar, Peshawar, PAK; 2 Surgery, Lady Reading Hospital Medical Teaching Institution (MTI) Peshawar, Peshawar, PAK; 3 Paediatrics, Lady Reading Hospital Medical Teaching Institution (MTI) Peshawar, Peshawar, PAK; 4 Emergency Medicine, Lady Reading Hospital Medical Teaching Institution (MTI) Peshawar, Peshawar, PAK; 5 Paediatrics, Ali Medical Center Peshawar, Peshawar, PAK

**Keywords:** neonatal care, risk factors, incidence, early preterm neonates, intraventricular hemorrhage

## Abstract

Background: Early preterm infants are susceptible to a serious disorder called intraventricular hemorrhage (IVH), which may cause severe neurological damage.

Objective: To determine the incidence of IVH in preterm infants at Lady Reading Hospital, Peshawar, Pakistan, and to identify associated risk factors and potential preventive measures.

Methodology: This cross-sectional research examined the prevalence of IVH among early preterm infants and was carried out at Lady Reading Hospital in Peshawar from 1 January 2021 to 31 December 2023. After excluding individuals with congenital defects, insufficient medical records, or non-consent, the research comprised 210 newborns born before 28 weeks of gestation and diagnosed with IVH during the first 72 hours of life. Medical record reviews and in-person observations were used to gather data, with an emphasis on clinical, risk, and demographic characteristics. Using the Statistical Package for the Social Sciences (IBM SPSS Statistics for Windows, IBM Corp., Version 25.0, Armonk, NY) with a significance threshold of p < 0.05, descriptive techniques were used in the statistical studies to summarize the features and inferential approaches, such as univariate and multivariate logistic regression, to identify IVH risk variables.

Results: Among the 210 early preterm newborns studied, the frequency of IVH according to severity was as follows: 79 infants (37.62%) had Grade I, 65 infants (30.95%) had Grade II, 39 infants (18.57%) had Grade III, and 27 infants (12.86%) had Grade IV. Three key demographic findings were that 63 births (30.00%) occurred before 26 weeks of gestation, 87 infants (41.43%) had birth weights of less than 1000 grams, and 111 infants (52.86%) were male. Significant predictors of IVH identified through multivariate logistic regression included birth weight less than 1000 grams (odds ratio (OR) = 3.10, 95% confidence interval (CI): 1.78-5.42, p < 0.01), gestational age less than 26 weeks (OR = 2.68, 95% CI: 1.50-4.76, p < 0.01), Apgar score ≤5 (OR = 4.01, 95% CI: 2.23-7.21, p < 0.01), resuscitation at birth (OR = 2.23, 95% CI: 1.12-4.45, p = 0.02), mechanical ventilation (OR = 3.55, 95% CI: 1.85-6.82, p < 0.01), and sepsis (OR = 2.98, 95% CI: 1.50-5.92, p = 0.02).

Conclusion: The high incidence of IVH and its association with critical risk factors underscore the need for improved neonatal care practices and targeted interventions in early preterm infants.

## Introduction

A dangerous and often severe disease that affects preterm newborns, especially those delivered before 28 weeks of gestation, is intraventricular hemorrhage (IVH) [1,2]. Fragile blood arteries in the brain's ventricles burst, causing IVH, which may cause serious neurological damage and developmental difficulties [3,4]. Because of their immature central nervous systems and vascular systems, early preterm infants have a noticeably higher prevalence of IVH [5]. The emphasis has progressively moved towards understanding and reducing the hazards associated with IVH in this susceptible group as newborn care procedures and survival rates have improved [6].

Calisici et al. found that out of 138 preterm infants diagnosed with severe IVH (Grades 3-4), 74 (53.6%) completed follow-up evaluations at 18-24 months' corrected age [7]. The incidence and consequences of IVH in early preterm neonates have not been thoroughly explored in Pakistan, a country with a notably high preterm birth rate [8]. There is a dearth of specialized research on the early preterm population in the area, despite some data being accessible from larger studies on preterm newborns [9]. According to recent research, there is a great deal of regional variation in IVH incidence rates, which is often caused by variations in newborn care methods and screening procedures [10]. Diverse incidence rates have been recorded in studies done in different locations, underscoring the need to do research relevant to a given place in order to comprehend local trends and advance worldwide understanding [11,12]. The healthcare system in Peshawar is confronted with distinct problems, such as limited resources and discrepancies in prenatal and neonatal treatment protocols, which might potentially impact the prevalence of IVH in premature neonates [13].

The precise knowledge of the occurrence of IVH in prematurely born infants in Peshawar is lacking in study. This gap highlights the need for a focused study to close the information gap in the area, evaluate the quality of newborn care already provided, and pinpoint particular risk factors relevant to the local environment. The project is to close this knowledge gap and provide insightful information that can improve newborn care protocols, guide clinical practices, and ultimately improve outcomes for this susceptible group.

Research objective

The objective of this study was to determine the prevalence of IVH among early preterm neonates at Lady Reading Hospital, Peshawar, Pakistan, and identify associated risk factors and potential preventive measures.

## Materials and methods

Study design and settings

A cross-sectional approach was used in this research to look at the occurrence of IVH in prematurely born infants. The study was carried out from 1 January 2021 to 31 December 2023 at Lady Reading Hospital in Peshawar, Pakistan. Because of the hospital's large patient traffic and comprehensive newborn care facility, which provide a representative sample of the early preterm neonate population in the area, this setting was selected. Diagnosis of IVH in this study was primarily based on cranial ultrasound, which is the standard and most widely used method for initial detection of IVH in preterm infants. Ultrasound imaging was performed within the first 72 hours of life to identify and grade the severity of IVH.

Inclusion and exclusion criteria

Babies who are diagnosed with IVH during the first 72 hours of life and whose parents or guardians have given their informed agreement to participate in the screening. Infants without full medical records, those whose parents or guardians declined their participation permission, and those whose congenital defects influence the frequency of IVH are not eligible.

Sample size

The sample size of 210 early preterm neonates was selected based on the hospital's annual birth records and the need for statistical precision in estimating the incidence rate of IVH. This sample size ensures adequate power to detect significant findings with a margin of error of ±5%. It represents a substantial subset of the population, balancing feasibility with the goal of achieving reliable and generalizable results. This number was determined considering practical constraints and ethical considerations, ensuring both robustness and manageability of the study.

Data collection

Data collection involved a review of neonatal medical records, including demographic information, clinical details, and outcomes. Intracranial hemorrhage was diagnosed using cranial ultrasound, the standard imaging modality for assessing IVH in neonates. IVH severity was classified into four grades: Grade I (isolated germinal matrix hemorrhage), Grade II (IVH without ventricular dilation), Grade III (hemorrhage with ventricular dilation), and Grade IV (hemorrhagic parenchymal involvement).

Apgar scores at one and five minutes were recorded to evaluate the immediate postnatal condition and guide resuscitative efforts. These scores helped determine the need for and effectiveness of resuscitation interventions, including positive pressure ventilation (PPV), chest compressions, and intubation. Additionally, data on prenatal factors (such as antenatal steroids and maternal hypertension), perinatal factors (like mode of delivery), and postnatal factors (including mechanical ventilation and sepsis) were collected to assess their influence on IVH risk.

Statistical analysis

Descriptive and inferential statistical techniques were used to examine the data. The demographic and clinical features of the newborns were compiled using descriptive statistics, such as means, standard deviations, and frequencies. The study computed the incidence rate of IVH and used univariate and multivariate logistic regression analysis to identify risk variables linked to the condition. The Statistical Package for the Social Sciences (IBM SPSS Statistics for Windows, IBM Corp., Version 25.0, Armonk, NY) was used for all statistical analyses, with a significance threshold of p < 0.05.

Ethical approval

The Institutional Review Board of Lady Reading Hospital Medical Teaching Institution issued approval 107/LRH/MTI, dated 19/12/2020. All participating infants' parents or guardians gave their informed permission. Ethics guidelines for research involving human participants were followed in this study, including the right to secrecy and the ability to leave the study at any time.

## Results

Table 1 presents the demographic features of the 210 neonates, revealing that 111 were male (52.86%), and 99 were female (47.14%). A total of 41.43% of birth weights (n = 87) weighed less than 1000 grams, 33.81% weighed between 1000 and 1499 grams (n = 71), and 24.76% weighed more than 1500 grams (n = 52). Thirty percent of babies were delivered before 26 weeks (n = 63), and 70.00% between 26 and 27+6 weeks (n = 147), according to gestational age. Around 33.81% of the sample (n = 71) scored ≤5 on the Apgar scale, whereas 66.19% of the sample (n = 139) scored >5. In 20.48% of instances (n=43), there were multiple births; in 79.52% of cases (n=167), there were single births. The distribution of maternal ages was 13.81% under 20 (n = 29), 69.52% between 20 and 35 (n = 146), and 16.67% over 35 (n = 35).

**Table 1:** Demographic Profile of Early Preterm Neonates (n=210)

Characteristics		Number of Patients (n)	Percentage (%)
Gender	Male	111	52.86
	Female	99	47.14
Birth Weight (grams)	<1000	87	41.43
	1000-1499	71	33.81
	≥1500	52	24.76
Gestational Age (weeks)	<26	63	30.00
	26 to 27+6	147	70.00
Apgar Score (at 5 minutes)	≤5	71	33.81
	>5	139	66.19
Multiple Births	Yes	43	20.48
	No	167	79.52
Maternal Age (years)	<20	29	13.81
	20-35	146	69.52
	>35	35	16.67

**Figure 1:**
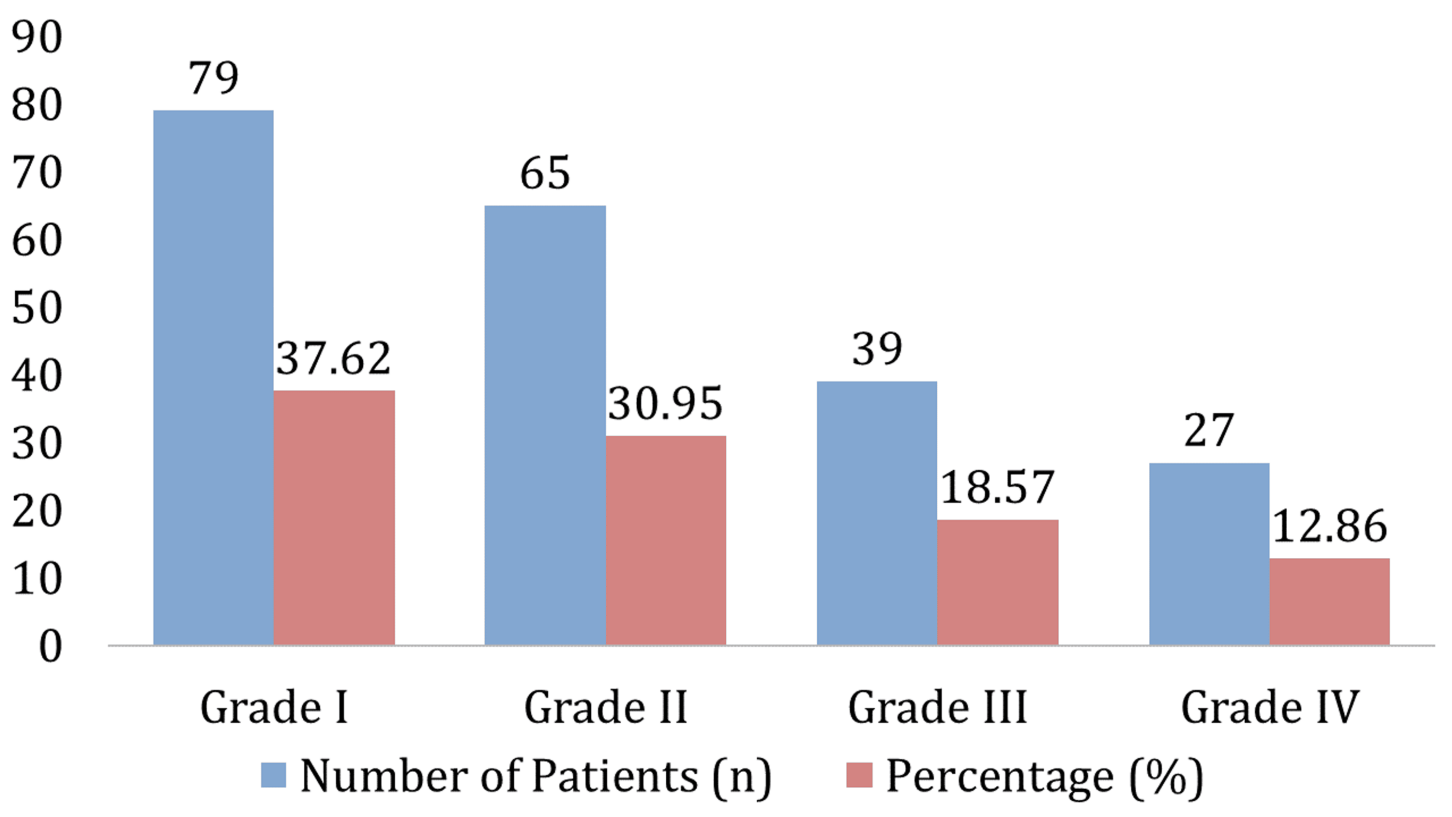
Incidence and Severity of IVH IVH: intraventricular hemorrhage

The frequency and severity of IVH in 210 preterm infants are shown in Figure 1. There are 79 patients with Grade I IVH (37.22% of the distribution), 65 patients with Grade II IVH (30.95%), 39 patients with Grade III IVH (18.57%), and 27 patients with Grade IV IVH (12.86%).

The distribution of prenatal variables among early preterm newborns (n = 210) is shown in Table 2. Prenatal steroids were given to most newborns; 154 patients (73.81%) of those got them, compared to 56 patients (26.19%) who did not. Of the newborns, 47 patients (22.38%) had maternal hypertension, whereas 163 patients (77.62%) did not have this disease. Of the newborns, 29 patients (13.81%) had maternal diabetes, whereas 181 patients (86.19%) did not have a diagnosis for this illness.

**Table 2:** Distribution of Prenatal Factors Among Early Preterm Neonates (n=210)

Factors		Number of Patients (n)	Percentage (%)
Antenatal Steroids	Yes	154	73.81
	No	56	26.19
Maternal Hypertension	Yes	47	22.38
	No	163	77.62
Maternal Diabetes	Yes	29	13.81
	No	181	86.19

The prenatal risk factors for early preterm neonates are listed in Table 3. A total of 118 (56.19%) of the 210 newborns had a cesarean section, while 92 newborns (43.81%) were delivered vaginally. Of the 210 early preterm neonates, 40 neonates (19.05%) received no resuscitation, while 120 neonates (57.14%) received PPV, 30 neonates (14.29%) required chest compressions, and 20 neonates (9.52%) were intubated at birth.

**Table 3:** Perinatal Factors Among Early Preterm Neonates (n=210)

Factors		Number of Patients (n)	Percentage (%)
Mode of Delivery	Vaginal	92	43.81
	Cesarean Section	118	56.19
Resuscitation at Birth	No Resuscitation	40	19.05
	Positive Pressure Ventilation (PPV)	120	57.14
	Chest Compressions	30	14.29
	Intubation	20	9.52

The distribution of postnatal variables in early preterm infants is shown in Table 4. A total of 127 (60.48%) of the 210 neonates needed invasive mechanical ventilation, while 83 neonates (39.52%) did not. Forty-nine newborns (23.33%) had sepsis, whereas 161 newborns (76.67%) had no such illness.

**Table 4:** Distribution of Postnatal Factors Among Early Preterm Neonates (n=210)

Factors		Number of Patients (n)	Percentage (%)
Mechanical Ventilation	Yes	127	60.48
	No	83	39.52
Sepsis	Yes	49	23.33
	No	161	76.67

The multivariate logistic regression analysis for IVH among early preterm newborns is shown in Table 5. Birth weight (<1000 grams vs. ≥1000 grams) has an odds ratio (OR) of 3.10 (95% confidence interval (CI): 1.78-5.42, p < 0.01), gestational age (<26 weeks vs. 26-27+6 weeks) of 2.68 (95% CI: 1.50-4.76, p < 0.01), Apgar score (≤5 vs. >5) has an OR of 4.01 (95% CI: 2.23-7.21, p < 0.01), resuscitation at birth (Yes vs. No) of 2.23 (95% CI: 1.12-4.45, p = 0.02), mechanical ventilation (Yes vs. No) of 3.55 (95% CI: 1.85-6.82, p < 0.01), and sepsis (Yes vs. No) of 2.98 (95% CI: 1.50-5.92, p = 0.02).

**Table 5:** Multivariate Logistic Regression Analyses for Intraventricular Hemorrhage Among Early Preterm Neonates (n=210)

Variables	Odds Ratio (OR)	95% Confidence Interval	p-value
Gender (Male vs. Female)	1.42	0.78-2.59	0.24
Birth Weight (<1000 grams vs. ≥1000 grams)	3.10	1.78-5.42	<0.01
Gestational Age (<26 weeks vs. 26 to 27+6 weeks)	2.68	1.50-4.76	<0.01
Apgar Score (≤5 vs. >5)	4.01	2.23-7.21	<0.01
Multiple Births (Yes vs. No)	1.85	0.99-3.45	0.05
Maternal Age (<20 years vs. 20-35 years)	1.60	0.68-3.75	0.27
Antenatal Steroids (Yes vs. No)	0.63	0.34-1.15	0.13
Maternal Hypertension (Yes vs. No)	1.74	0.89-3.40	0.11
Maternal Diabetes (Yes vs. No)	1.23	0.56-2.71	0.61
Mode of Delivery (Cesarean vs. Vaginal)	1.27	0.71-2.27	0.42
Resuscitation at Birth (Yes vs. No)	2.23	1.12-4.45	0.02
Mechanical Ventilation (Yes vs. No)	3.55	1.85-6.82	<0.01
Sepsis (Yes vs. No)	2.98	1.50-5.92	0.02

## Discussion

This research found a noteworthy frequency of IVH in early preterm newborns: 37.62% of the neonates had Grade I IVH, 30.95% had Grade II IVH, 18.57% had Grade III IVH, and 12.86% had Grade IV IVH. These results are consistent with other research, which indicates that IVH is very prevalent in this susceptible group [14,15]. Furthermore, research by Calisici et al. [7] discovered that severe IVH affected around 20% of very preterm newborns, suggesting that our results are in line with the severity range seen worldwide. Our data, however, indicates a reduced percentage of severe cases, which may be due to regional variations in the methods and success rates of newborn treatment.

A substantial risk factor for IVH, 41.43% of newborns had a birth weight of less than 1000 grams. Our results are corroborated by a recent research by Klinger et al. [16] who found a correlation between a greater frequency of IVH and lower birth weights. This link is further highlighted by the OR for birth weight (<1000 grams vs. ≥1000 grams), which is 3.10 (95% CI: 1.78-5.42, p < 0.01), supporting results from related research that found similar risk ratios [17].

In terms of gestational age, 30.00% of newborns were born before 26 weeks, and the OR for IVH was substantially higher in this group (2.68; 95% CI: 1.50-4.76, p < 0.01). This finding aligns with other studies that show very preterm newborns had a greater risk of IVH than infants born later [18]. This emphasizes how crucial gestational age is in determining the risk of IVH.

With 33.81% of newborns scoring ≤5 at five minutes, the influence of Apgar scores on IVH was clearly seen. The IVH OR in this group is 4.01 (95% CI: 2.23-7.21, p < 0.01), which is consistent with other studies that found a correlation between low Apgar ratings and a higher risk of IVH [19]. This supports the idea that IVH may be predicted by the immediate postnatal state.

Furthermore, the results show that mechanical ventilation was necessary for 60.48% of neonates, and the hazard ratio for IVH was 3.55 (95% CI: 1.85-6.82, p < 0.01). This result is consistent with that of the earlier research [20], which found that mechanical ventilation was a substantial risk factor for IVH. The idea that severe beginning circumstances increase the incidence of IVH is further supported by the high prevalence of IVH (80.95%) among individuals who required resuscitation at delivery.

These findings demonstrate the need for focused therapies for newborns that require acute postnatal care, have low birth weights, low Apgar scores, or are very preterm. Effective preventive and care methods for IVH in preterm infants must be tailored via ongoing research and targeted investigations.

Study limitations

This study has several limitations related to its retrospective design. The reliance on historical medical records introduces potential issues with missing data and inaccuracies, which may affect the reliability of the findings. The cross-sectional nature of the study also limits our ability to establish temporal relationships or causation between risk factors and IVH. The study’s focus on a single hospital in Peshawar may reduce the generalizability of the results to other settings or populations.

To address these limitations, future research should consider employing longitudinal and multicenter approaches. Longitudinal studies could provide insights into causal relationships and changes over time, while multicenter studies would enhance the generalizability of the findings and help to identify broader patterns and risk factors associated with IVH. Implementing rigorous data validation procedures and standardizing record-keeping practices across institutions could also mitigate some of the biases and inaccuracies associated with retrospective data collection.

## Conclusions

This research indicates that early preterm neonates at Lady Reading Hospital in Peshawar exhibit a high prevalence of IVH, with varying degrees of severity. These findings are consistent with international studies, suggesting that the prevalence of IVH in this cohort is comparable to global trends, yet highlights important local variations in neonatal care. The study identifies several key risk factors for IVH, including very low birth weight, severe prematurity, low Apgar scores, and the necessity for invasive mechanical ventilation. These factors align with those documented in international research but underscore specific considerations relevant to the local context. In addition to identifying risk factors, it is crucial to consider the potential adverse neurodevelopmental outcomes associated with IVH. Such outcomes may include cognitive, motor, and sensory impairments, which could have significant long-term consequences for affected neonates. Addressing these risks requires not only targeted interventions to improve immediate neonatal care but also ongoing research to develop and implement effective preventative and management strategies.

Our data highlight the urgent need for focused interventions and more localized research to enhance both prevention and management techniques, ultimately aiming to improve outcomes for this vulnerable population. Effective strategies should include enhanced prenatal care, optimized delivery practices, and comprehensive postnatal interventions. Continued efforts in research and clinical practice are essential to mitigate IVH risk factors and improve the overall care for early preterm neonates.
